# Supplementary material for: Notch transactivates Rheb to maintain the multipotency of TSC-null cells
Source: Nat Commun. 2017 Nov 29;8:1848. doi: 10.1038/s41467-017-01845-1 (PMC5705704; doi:10.1038/s41467-017-01845-1)
Supplement: Supplementary file 1 — Supplementary information [file 41467_2017_1845_MOESM1_ESM.pdf]

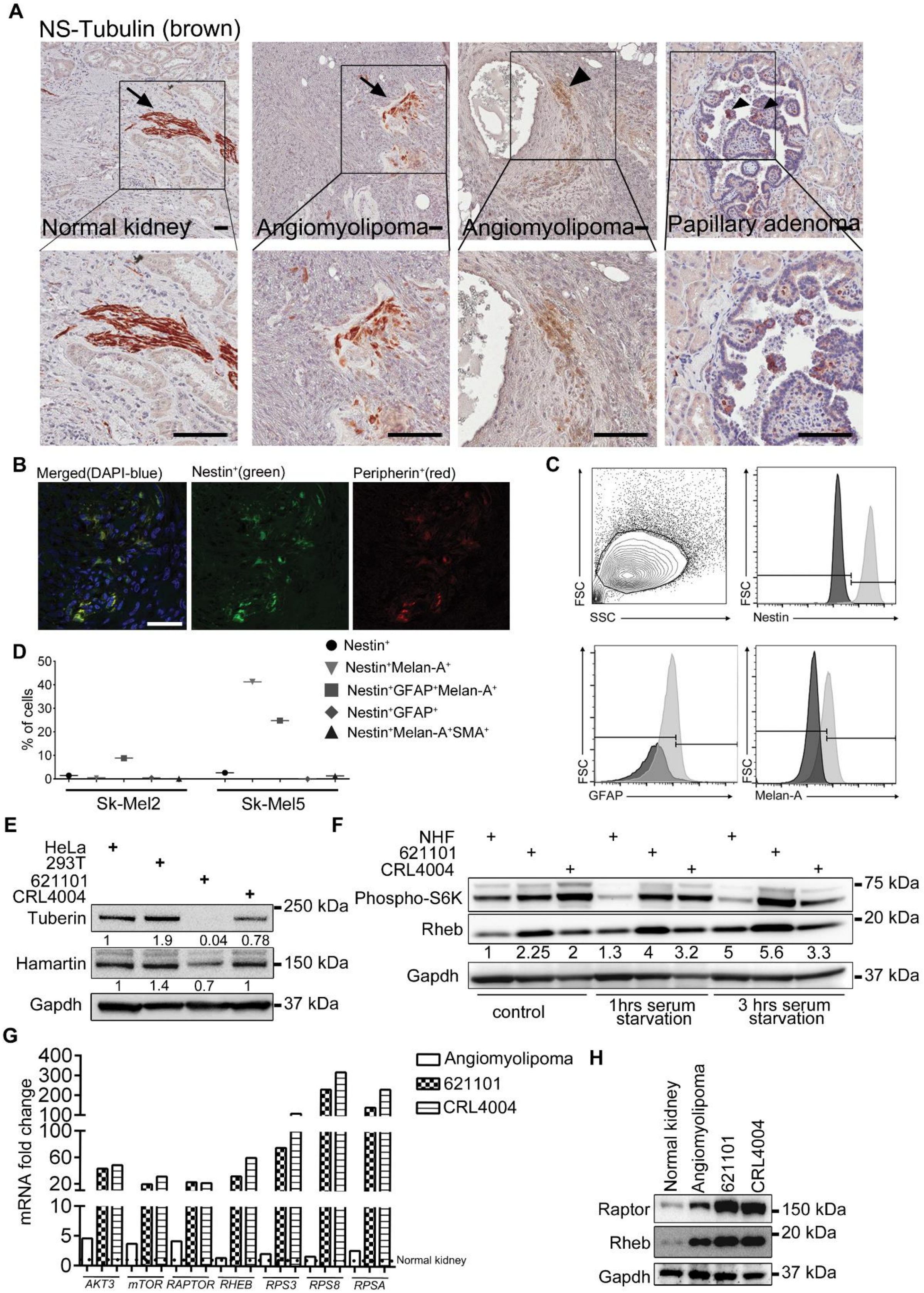
A
NS-Tubulin (brown)
Normal kidney
Angiomyolipoma
Angiomyolipoma
Papillary adenoma
B
Merged(DAPI-blue)
Nestin+(green)
Peripherin+(red)
C
FSC
SSC
Nestin
GFAP
Melan-A
D
% of cells
Nestin+
Nestin+Melan-A+
Nestin+GFAP+Melan-A+
Nestin+GFAP+
Nestin+Melan-A+SMA+
Sk-Mel2
Sk-Mel5
E
HeLa
293T
621101
CRL4004
Tuberin
250 kDa
1 1.9 0.04 0.78
Hamartin
150 kDa
1 1.4 0.7 1
Gapdh
37 kDa
F
NHF
621101
CRL4004
Phospho-S6K
75 kDa
Rheb
20 kDa
1 2.25 2 1.3 4 3.2 5 5.6 3.3
Gapdh
37 kDa
control
1hrs serum starvation
3 hrs serum starvation
G
mRNA fold change
Angiomyolipoma
621101
CRL4004
Normal kidney
AKT3
mTOR
RAPTOR
RHEB
RPS3
RPS8
RPSA
H
Normal kidney
Angiomyolipoma
621101
CRL4004
Raptor
150 kDa
Rheb
20 kDa
Gapdh
37 kDa

Supplementary Figure 1. Differentiation abnormalities and increased expression of Rheb in angiomyolipoma, 621-101 and CRL4004 angiomyolipoma-derived cells.

(**A**) NS-tubulin expression (brown) in nerves of the normal kidney, arrows (first panel, arrow); sporadic angiomyolipoma (second panel, arrow; third panel, arrowhead), and the papillary lesion within kidney adjacent to angiomyolipoma (fourth panel, arrowheads). Lower panels: histological details within the areas marked by the corresponding inserts in the upper panels.

(**B**) Nestin (green) and peripherin (red) expression in angiomyolipoma by immunofluorescence; yellow: co-expression of both molecules, blue-DAPI. (**C and D**) FACS analysis of differentiation markers in angiomyolipoma or melanoma-derived cells; (**C**) Cells were gated based on forward (FSC) and side scatter (SSC) properties (contour plot, left panel), and percentages of cells expressing individual markers were determined by comparing antibody stained samples (light grey) to samples stained with isotype control IgG (dark gray histograms, three left panels). (**D**) FACS analysis of the differentiation markers in melanoma cell lines (Sk-Mel2 and Sk-Mel5) grown in standard RPMI medium. Percentages of cells expressing nestin alone or co-expressing differentiation markers. (**E**) Western blot analysis of hamartin and tuberin in HeLa, 293T, 621-101 and CRL4004 angiomyolipoma cells. Numeric values represent densitometry analysis of the expression of tuberin or hamartin relative to expression of Gapdh. (**F**) Rheb and phospho-S6K in normal human fibroblasts (NHFs), 621-101 and CRL4004 angiomyolipoma cells in the presence or absence of serum by Western immunoblotting. Numeric values represent densitometry analysis of the expression of Rheb relative to expression of Gapdh. (**G**) q(RT)-PCR of *AKT3*, *mTOR*, *RAPTOR*, *RHEB*, *RPS3*, *RPS8* and *RPSA* in sporadic angiomyolipoma, 621-101 and CRL4004 angiomyolipoma-derived cells relative to the normal kidney from the same patient. The expression of mTOR signaling genes was normalized to *GAPDH*. Candidate genes were chosen based on

RNA-Seq analyses. (**H**) Rheb and Raptor in angiomyolipoma, the normal kidney adjacent to angiomyolipoma, 621-101 and CRL4004 angiomyolipoma cells by Western immunoblotting. Scale bar: 50 μm.

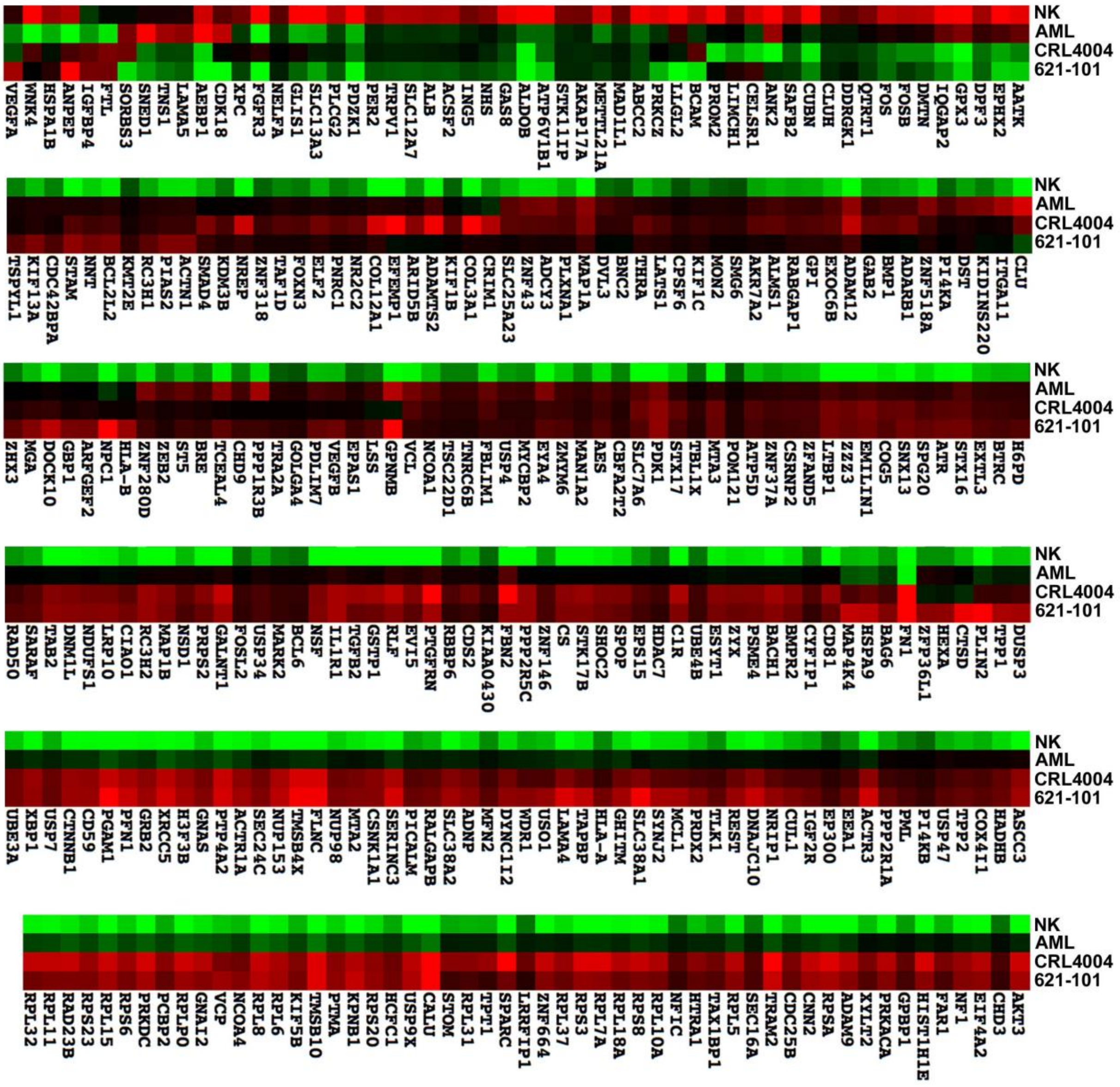

Supplementary Figure 2. The gene expression patterns shown as a heatmap, in the normal kidney adjacent to angiomyolipoma, angiomyolipoma from the same patient, 621-101 and CRL4004 angiomyolipoma-derived cells.

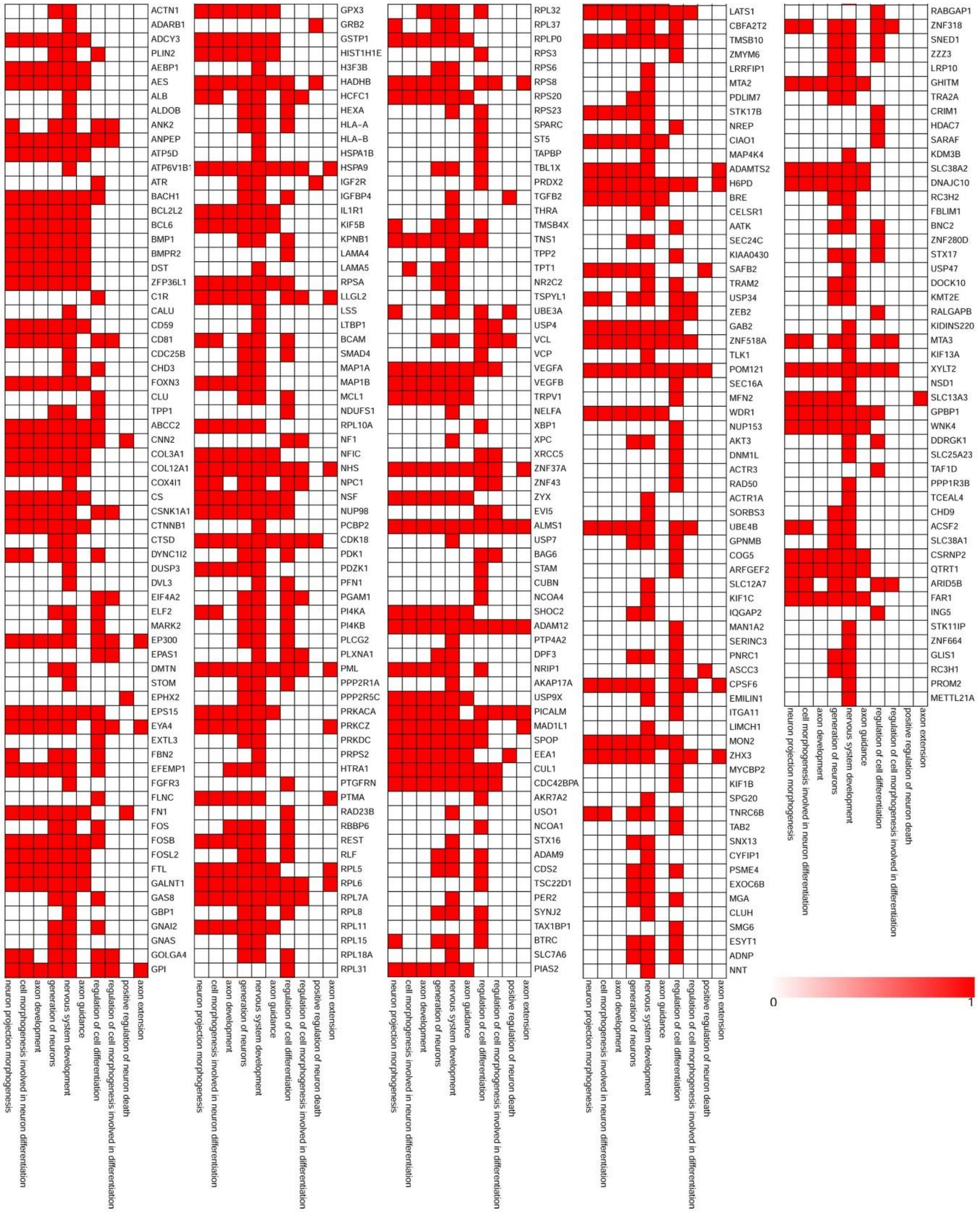

Supplementary Figure 3. Gene Ontology enrichment analysis (GOEA) of transcriptomic profile of sporadic angiomyolipoma, 621-101 and CRL4004 angiomyolipoma-derived cells in

comparison to the normal kidney from the same patient. Three gene lists were intersected, and only the common genes, which were differentially expressed in angiomyolipoma, angiomyolipoma-derived cells, vs. normal kidney (~1400) were used for the gene ontology enrichment analysis. The red denotes genes (shown in row) within corresponding significantly enriched categories (shown in column).

Fig S4

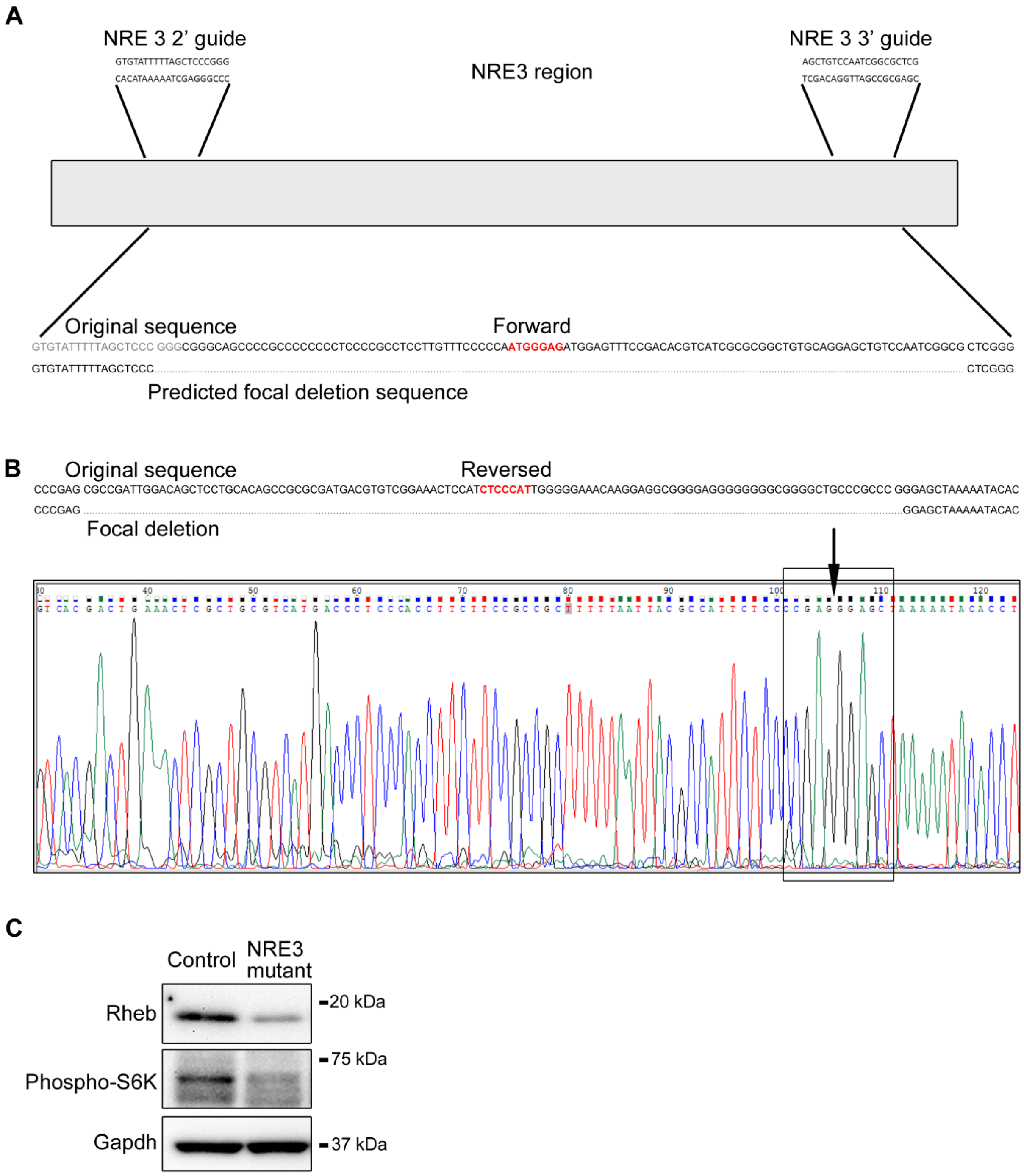

Supplementary Figure 4. Deletion of NRE3 region from the endogenous *RHEB* promoter suppresses expression and activation of Rheb in angiomyolipoma cells.

(**A**) Scheme of CRISPR/Cas9-mediated mutagenesis of the Rheb promoter region in CRL4004 angiomyolipoma cells. NRE3 sense and anti-sense guide sequences are shown in grey and NRE3 site is shown in red. Dotted line indicates predicted focal deletion within Rheb promoter; (**B**) Upper panel: reversed sequence of targeted NRE3 region blast against reversed sequence of CRL4004 angiomyolipoma cells. Dotted line indicate focal deletion of 107 bp containing NRE3 site (shown in red). Lower panel: original reversed sequence showing deletion of NRE3 region in CRL4004 cells; boxed sequence indicate ligation of truncated ends; (**C**) Expression of endogenous Rheb and phospho-S6K by Western immunoblotting in control and the NRE3 deleted CRL4004 cells (NRE3 mutant);

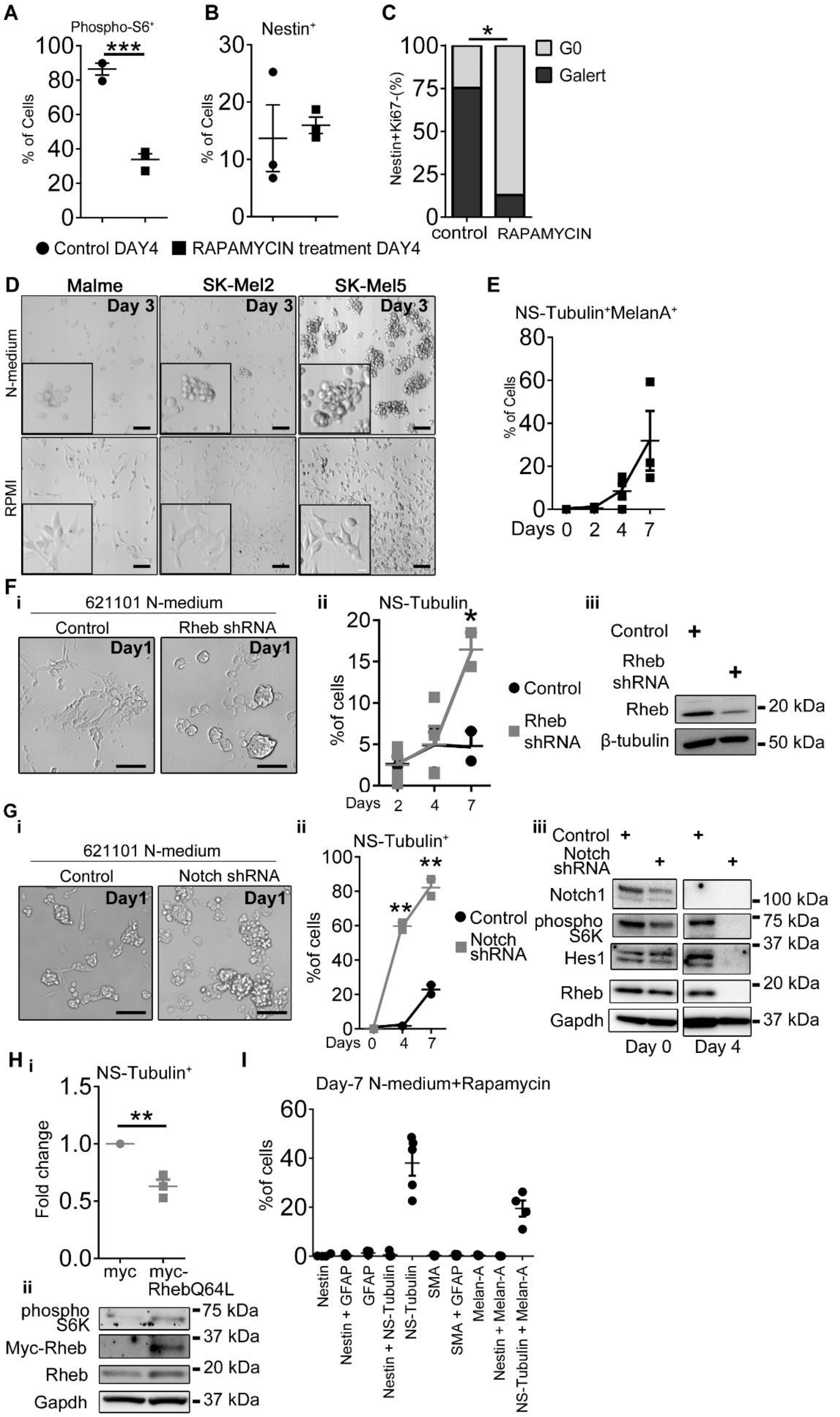

A
Phospho-S6+
% of Cells
B
Nestin+
% of Cells
C
Nestin+Ki67-(%)
G0
Galert
control
RAPAMYCIN
Control DAY4
RAPAMYCIN treatment DAY4
D
Malme
SK-Mel2
SK-Mel5
Day 3
N-medium
RPMI
E
NS-Tubulin+MelanA+
% of Cells
Days 0 2 4 7
F
i
621101 N-medium
Control
Rheb shRNA
Day1
ii
NS-Tubulin
%of cells
Days 2 4 7
Control
Rheb shRNA
iii
Control
Rheb shRNA
Rheb
β-tubulin
20 kDa
50 kDa
G
i
621101 N-medium
Control
Notch shRNA
Day1
ii
NS-Tubulin+
%of cells
Days 0 4 7
Control
Notch shRNA
iii
Control
Notch shRNA
Notch1
phospho S6K
Hes1
Rheb
Gapdh
100 kDa
75 kDa
37 kDa
20 kDa
37 kDa
Day 0
Day 4
H i
NS-Tubulin+
Fold change
myc
myc-RhebQ64L
ii
phospho S6K
Myc-Rheb
Rheb
Gapdh
75 kDa
37 kDa
20 kDa
37 kDa
I
Day-7 N-medium+Rapamycin
%of cells
Nestin
Nestin + GFAP
GFAP
Nestin + NS-Tubulin
NS-Tubulin
SMA
SMA + GFAP
Melan-A
Nestin + Melan-A
NS-Tubulin + Melan-A

Supplementary Figure 5. Rapamycin insensitive multipotent properties and neuronal differentiation of angiomyolipoma cells

(**A-C**) FACS analysis of phospho-S6, nestin and Ki67 in 621-101 cells on day 4 treated with rapamycin *vs.* DMSO (control); percentage of (**A**) phospho-S6 or (**B**) nestin positive cells. (**C**) Percentage of quiescent neural stem-like angiomyolipoma cells in G_0 (nestin$^+$Ki67$^-$phospho-S6$^-$) *vs.* G_{alert} (nestin$^+$Ki67$^-$phospho-S6$^+$). (**D**) Brightfield images of melanoma cells in RPMI or N-medium. (**E**) Percentages of angiomyolipoma-derived 621-101 cells expressing NS-tubulin and melan-A at different time points in N-medium by FACS. (**F**) (**i**) Brightfield images of control and Rheb shRNA-treated 621-101 cells in N-medium; (**ii**) Percentage of cells expressing NS-tubulin treated with control or Rheb shRNA (FACS); Rheb by Western immunoblotting in cells treated as in ii. (**G**) (**i**) Brightfield images of control and Notch shRNA-treated 621-101 cells in N-medium; (**ii**) Percentage of cells expressing NS-tubulin treated with control or Notch shRNA (FACS); (**iii**) Notch1, phopsho-S6K, Hes1 and Rheb by Western immunoblotting in cells treated as in ii. (**H**) (**i**) FACS analysis of the differentiation markers in CRL4004 angiomyolipoma cells expressing Q64L Rheb grown in N-medium for 24 hours; Fold change in percentages of CRL4004 cells expressing NS-tubulin only at day 2; (**ii**) Expression of myc-tagged Q64L Rheb and phospho-S6K at day 2 by Western immunoblotting; (**I**) FACS analysis of the differentiation markers in angiomyolipoma-derived cells grown in N-medium in the presence of rapamycin at day 7. Percentage of 621-101 cells expressing NS-tubulin alone or NS-tubulin and melan-A did not differ from untreated cells shown in **Fig. 3C-i-ii and Supplementary Fig. 4I**.

Data represent mean±s.e.m. Error bars are defined as means+s.e.m. *P≤0.05; **P≤0.01; ***P≤0.001; t-test; and are representative of three independent experiments with (A-I), n=4 (I), n=3 (A-F,H), n=2 (G). Scale bar: 50 µm.

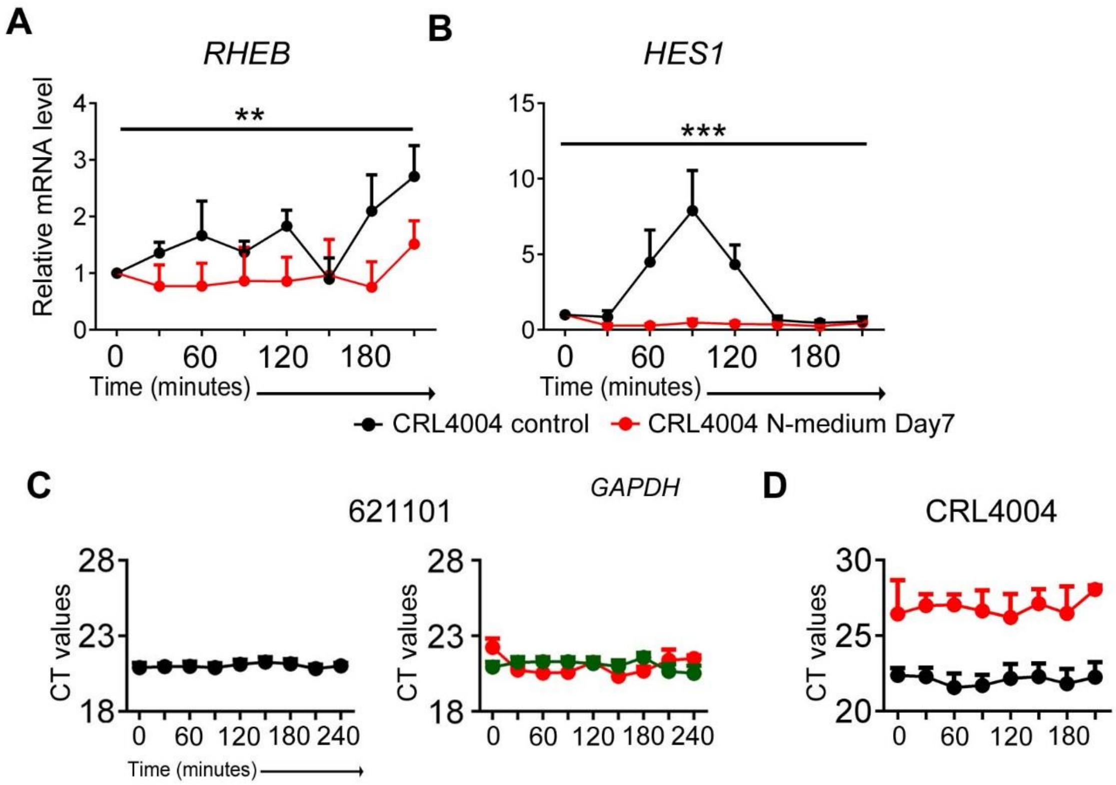

Supplementary Figure 6. Suppression of Rheb and Hes1 oscillation associates with neuronal differentiation of CRL4004 angiomyolipoma cells.

(**A-B**) q(RT)-PCR analysis of (**A**) *RHEB* and (**B**) *HES1* expression relative to *GAPDH* in synchronized CRL4004 AML cells grown in N-medium *vs.* DMEM (control, data from DMEM are also used in Figure 2A-iii-vi, 240 min time point is not shown here). (**C-D**) CT values of *GAPDH* that were used to determine the expression of *RHEB* and *HES1* in (**C**) 621-101 or (**D**) CRL4004 cells in DMEM (black) or N-medium (stimulated with FBS [green] or chick embryo extract [red]).

Data represent mean ± s.e.m. Error bars are defined as means+s.e.m. **P≤0.01; ***P≤0.001; two-way ANOVA; Data are representative of two independent experiments with n=3 (A-D).

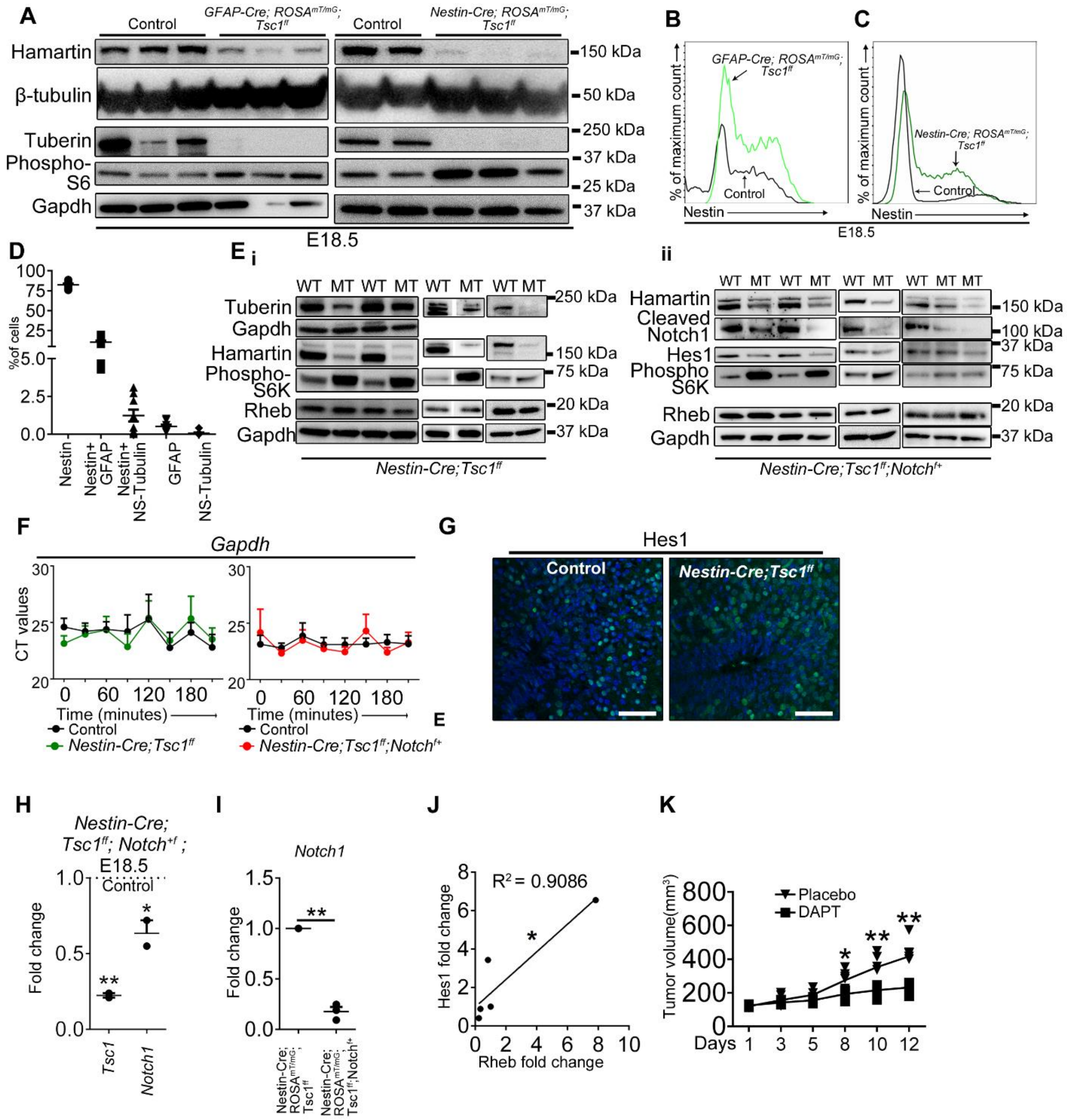

Supplementary Figure 7. Loss of *Tsc1/2* increases the number of neural stem cells and blocks cell differentiation *in vivo.*

(**A**) Western blot analysis of hamartin, tuberin and phospho-S6 in *GFAP-Cre;ROSA*$^{mT/mG}$*;Tsc1*^{ff} and *Nestin-Cre;ROSA*$^{mT/mG}$*;Tsc1*^{ff} mouse embryos. Data are representative of 11 randomly

selected mouse embryos from each group. (**B-C**) FACS analysis of nestin in neural tube (NT) cells of (**B**) *GFAP-Cre;ROSA*$^{mT/mG}$ (control, n=3) *vs. GFAP-Cre;ROSA*$^{mT/mG}$*;Tsc1*^{ff} (n=7) mouse embryos or (**C**) *Nestin-Cre;ROSA*$^{mT/mG}$ (control, n=5) *vs. Nestin-Cre;ROSA*$^{mT/mG}$*;Tsc1*^{ff} (n=5) mouse embryos. Histograms represent percentage of EGFP^{+}nestin^{+} at E18.5. (**D**) FACS analysis of the differentiation markers in cultured NT-derived cells isolated from *Nestin-Cre;Tsc1*^{ff} mouse embryo at E15.5; (data are representative of n=9 from two independent experiments). (**E**) Western blot analysis of hamartin, tuberin and phospho-S6K, Hes1, Rheb, cleaved Notch (Val1744) in NT cells of (**i**) *Nestin-Cre;Tsc1*^{ff} and (**ii**) *Nestin-Cre;Tsc1*^{ff}; *Notch1*$^{f+}$ vs. littermate control mouse embryos. Data are representative of n=8 (E-i) and n=9 (E-ii) randomly selected mouse embryos from three independent experiments. (**F**) CT values of *Gapdh* used to determine relative expression of *Rheb* and *Hes1* shown in Figure 6A and 6B by q(RT)-PCR in E15.5 littermate controls or *Nestin-Cre;Tsc1*^{ff}, or *Nestin-Cre;Tsc1*^{ff}*;Notch*$^{f+}$ embryos. (**G**) Immunofluorescence of Hes1 in NT cells from E17.5 *Nestin-Cre;Tsc1*^{ff} or *Nestin-Cre;Tsc1*^{ff}*;Notch*$^{f+}$ or littermate controls. Data are representative of 4 randomly selected mouse embryos from each group. (**H-I**) q(RT)-PCR analysis of *Tsc1* and/or *Notch1* in *Nestin-Cre;Tsc1*^{ff}, *Nestin-Cre;Tsc1*^{ff}*;Notch*$^{f+}$, *Nestin-Cre;ROSA*$^{mT/mG}$*;Tsc1*^{ff} or *Nestin-Cre;ROSA*$^{mT/mG}$*;Tsc1*^{ff}*;Notch*$^{f+}$ relative to littermate control embryos. (**J-K**) **Hes1 and Rheb expression in *Tsc2*-null tumors;** (**J**) Positive correlation between *Hes1* and *Rheb* expression in *Tsc2*-null ELT3 xenograft tumors assessed by q(RT)-PCR analysis of endogenous *Hes1* and *Rheb* mRNA relative to *Gapdh*. (**K**) Growth of *Tsc2*-null ELT3 cell xenograft tumors in mice treated with DAPT (n=5) or placebo control (n=6). Data represent mean tumor volume (mm^{3}) ± SEM; *P≤0.05; **P≤0.01; t-test.

Data represent mean ± s.e.m. Error bars are defined as means+s.e.m. *$P \leq 0.05$; **$P \leq 0.01$, one-sample t-test. Scale bar: 50 µm.

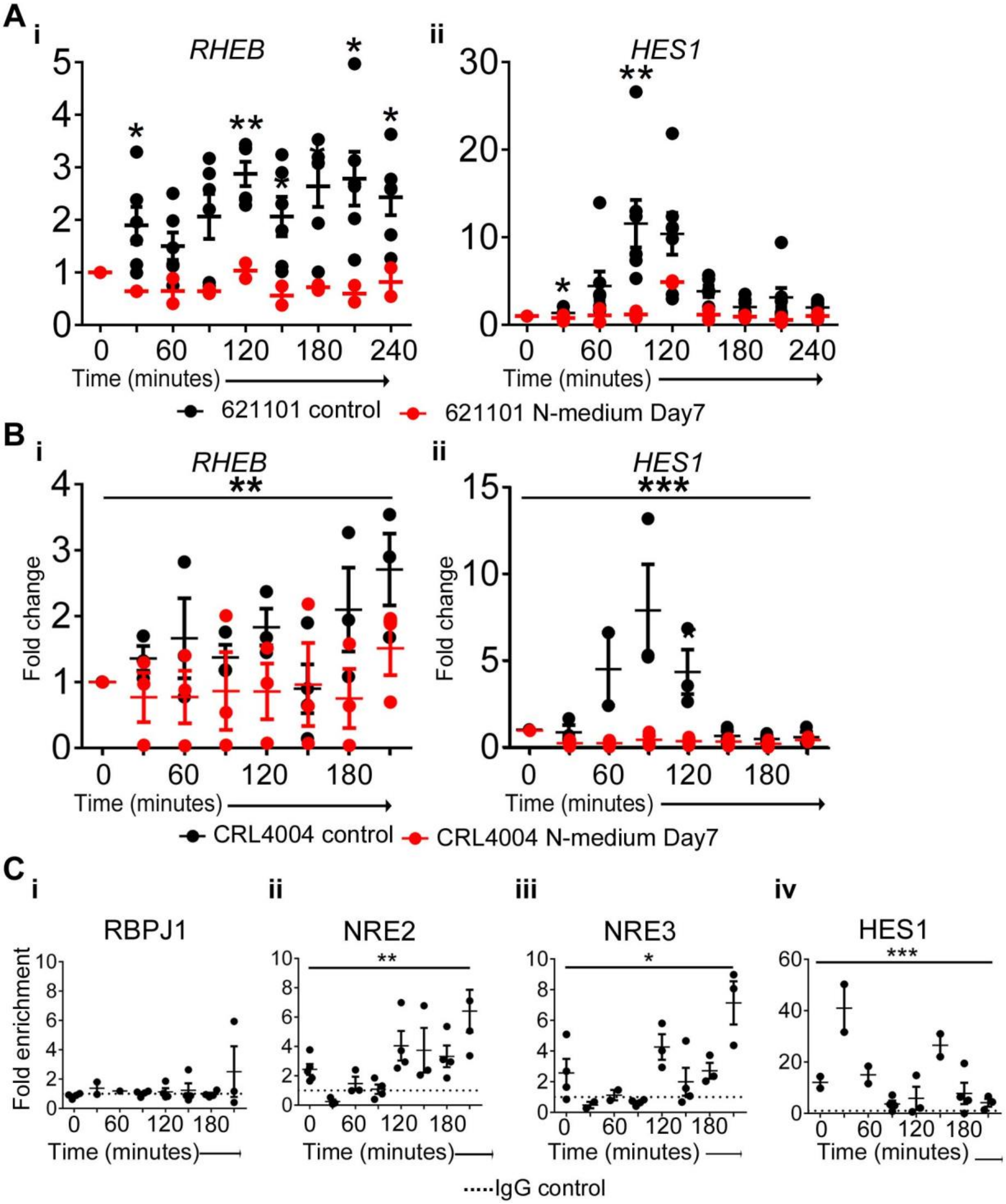

Supplementary Figure 8. *RHEB* and *HES1* oscillation shown as dot plots

(**A**) q(RT)-PCR of (**i**) *RHEB* and (**ii**) *HES1* relative to *GAPDH* in synchronized 621-101 cells in N-medium *vs.* DMEM (control, data as dot plots represent graphs shown in Figure 2A and 4A).

(**B**) q(RT)-PCR of (**i**) *RHEB* and (**ii**) *HES1* relative to GAPDH in synchronized 4004 cells in N-medium *vs.* DMEM (control, data as dot plots represent graphs shown in Figure 2A-iii-iv and S6). (**C**) Binding of Notch1 to the (**i**) potential RBPJ1, (**ii**) NRE2 and (**iii**) NRE3 within Rheb or the (**iv**) Hes1 promoter in synchronized 621-101 cells in DMEM by ChIP-qPCR as in 2B-ii-iii (data as dot plots represent graphs shown in Figure 2E and 4B).

Data represent means± s.e.m. Error bars are defined as means+s.e.m. *P≤0.05, **P≤0.01, ***P≤0.001; t-test (A), two-way ANOVA (B, C).

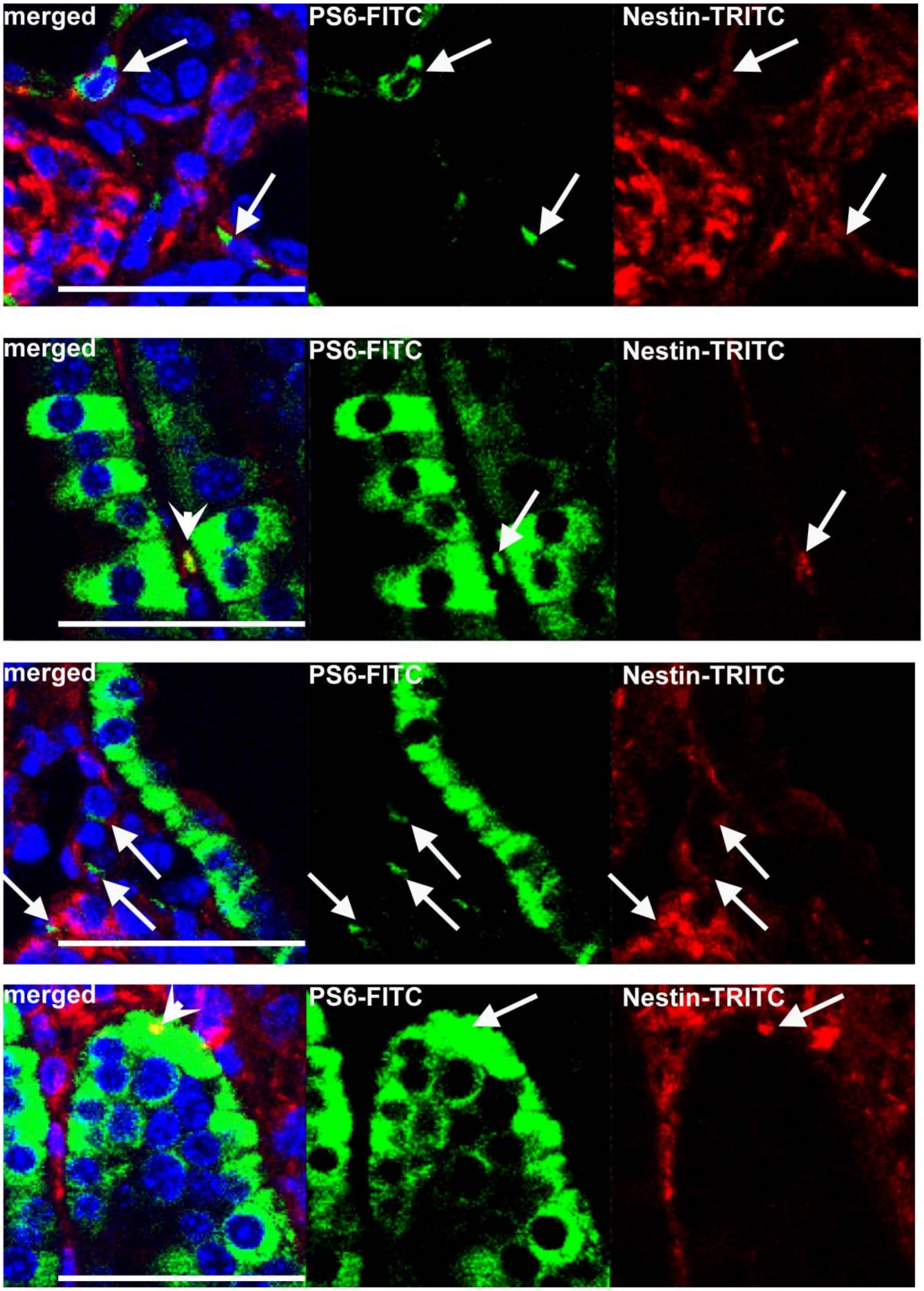

Supplementary Figure 9. Phospho-S6 in nestin expressing tumor cells

High power images, acquired using confocal microscopy, representing single layer from a Z-stack showing phospho-S6 (green, arrows) in nestin positive (red, arrows) tumor cells. Arrowheads indicate co-localization of nestin and phospho-S6 within the same region of the cytoplasm, while arrows indicate the presence of phopsho-S6 within the same nestin-positive cells, however, without evident co-localization.

Supplementary Figure 10. Uncropped western blot images

Uncropped western immunoblots relevant to Figure 1

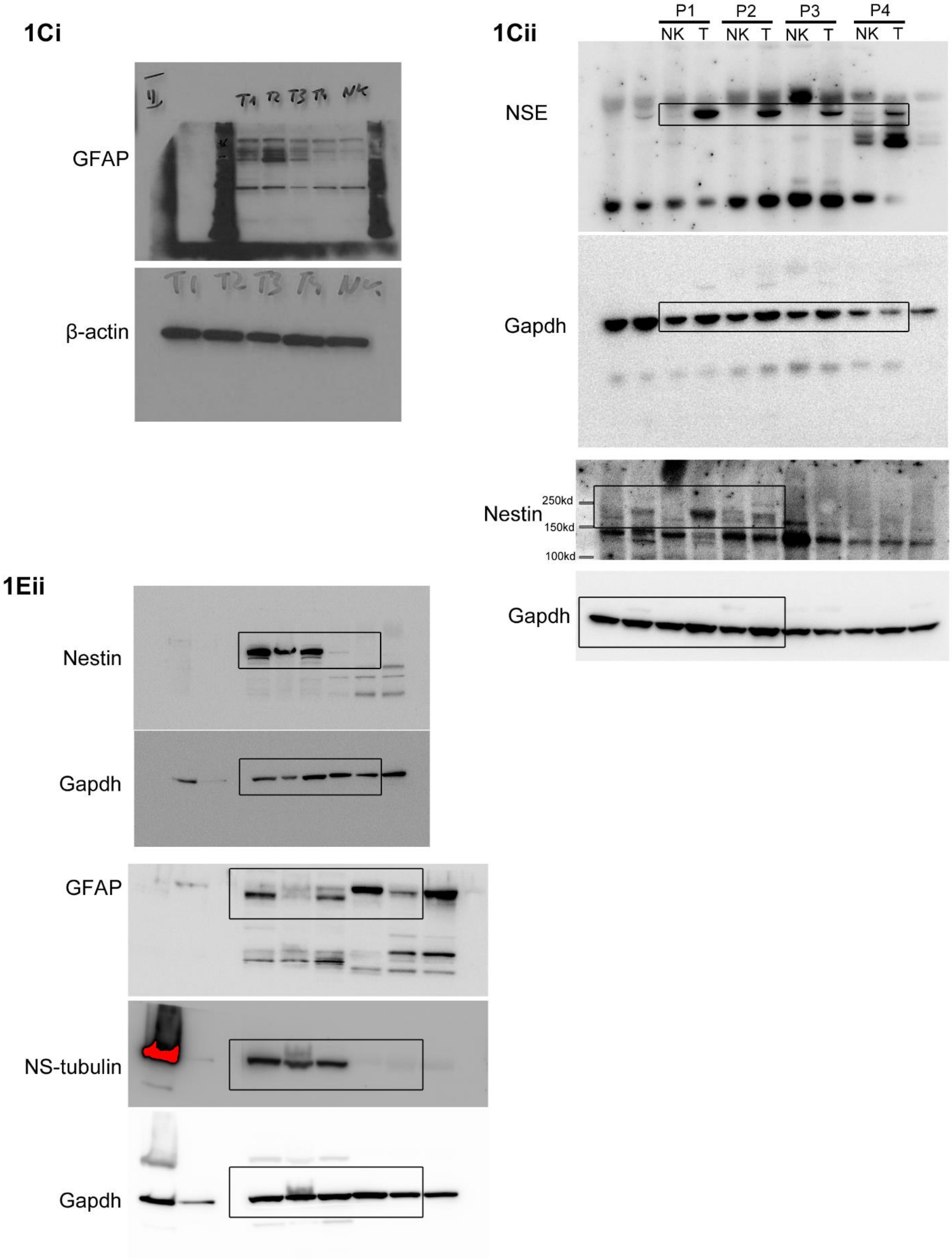

Uncropped western immunoblots relevant to Figure 2

2B

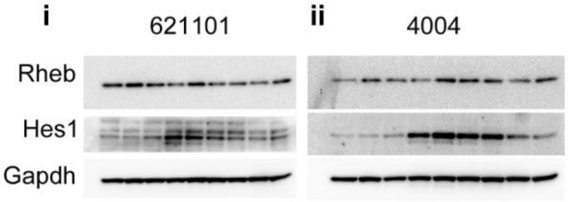

2D ii

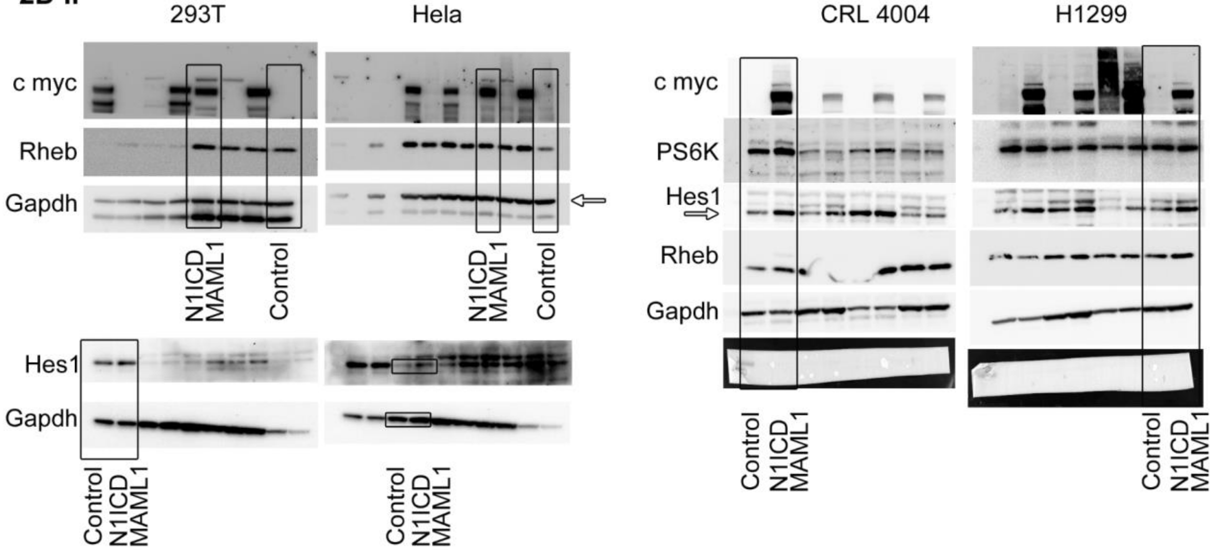

2Diii

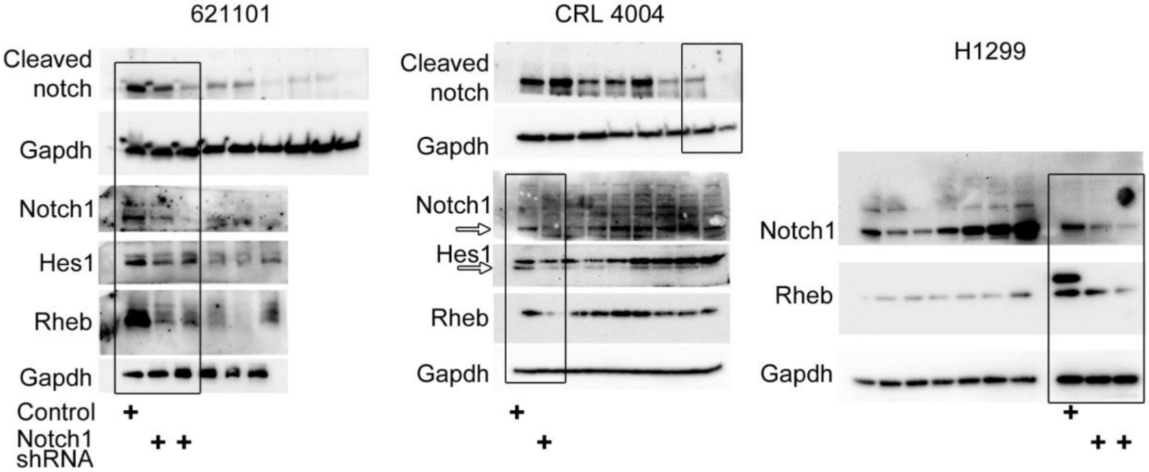

Uncropped western immunoblots relevant to Figure 4

4D

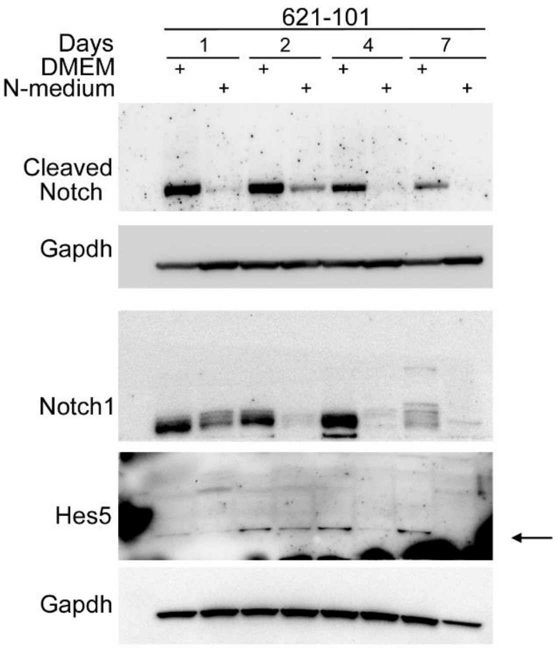

4G

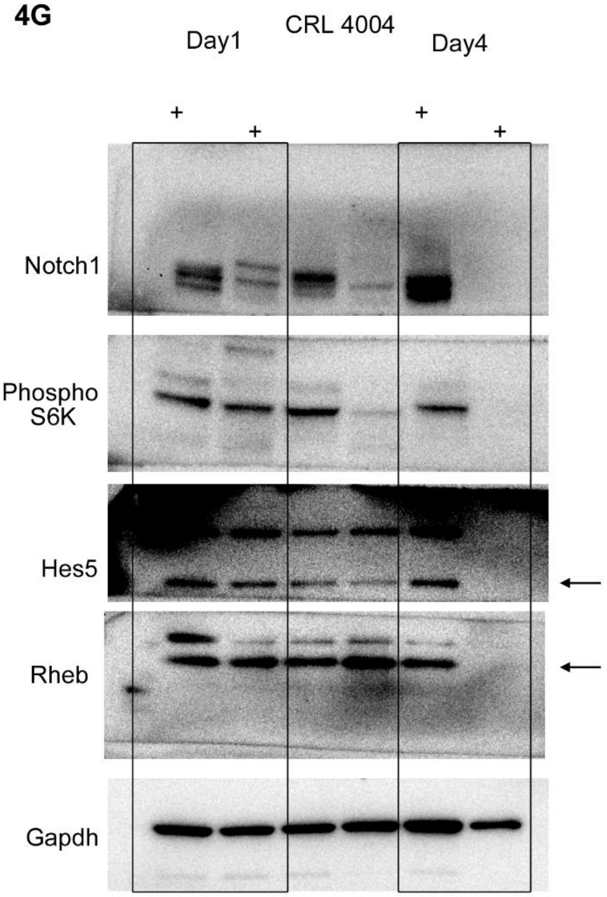

4G

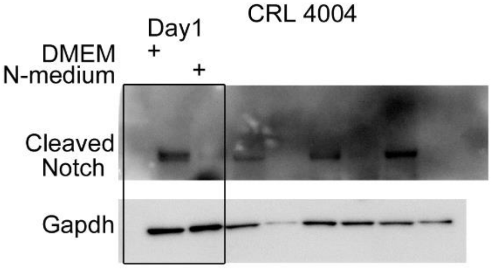

4G

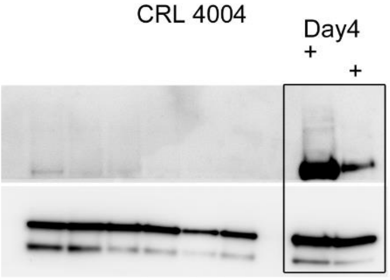

Uncropped western immunoblots relevant to Figure 6

6C

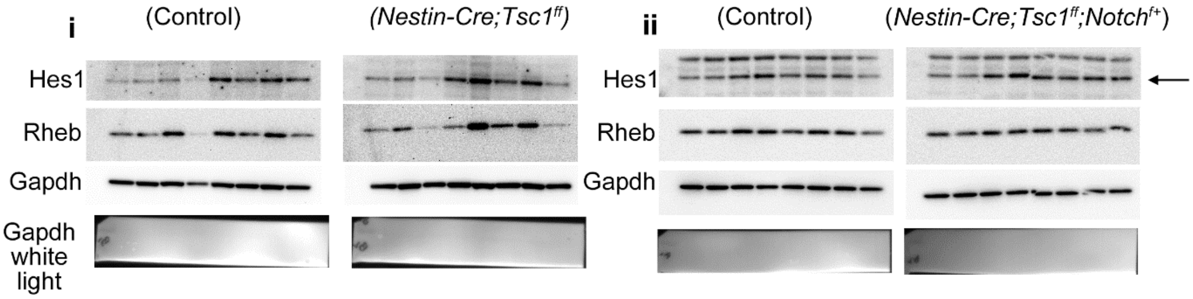

6Eii

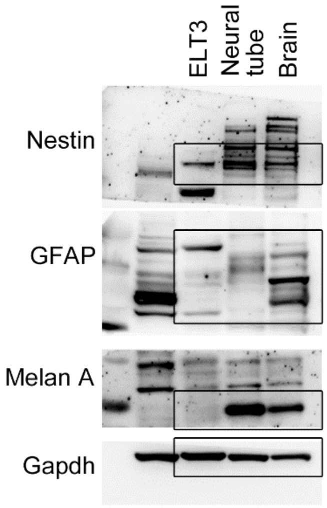

6Fiv

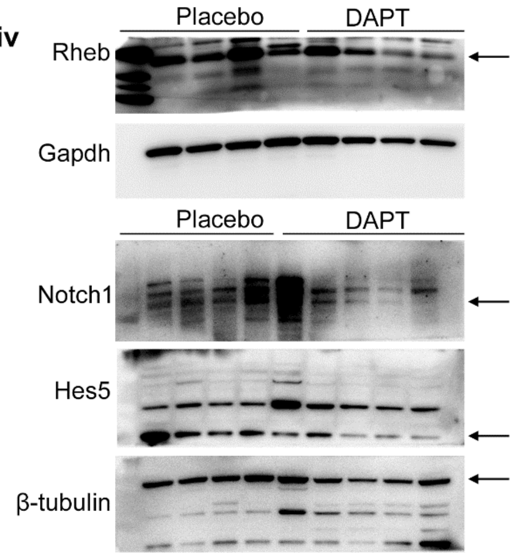

Supplementary Tables

Supplementary Table 1. Quantification of peripherin and nestin co-localization in LAM and angiomyolipoma patients.

LAM patient	Percentage of area expressing peripherin and/or nestin		
	Peripherin	Nestin	Nestin and peripherin co-localization
9043	42.02	29.71	4.12
9061	27.48	34.95	2.36
9036	35.25	32.89	3.10
9028	11.82	42.41	0.70
9039	45.59	30.81	6.09
9027	26.02	37.08	2.33
9032	33.64	31.90	3.92
9022	25.26	44.15	3.07
9030	34.52	36.88	4.56
**angiomyolipoma patient**	**Percentage of area expressing peripherin and nestin**		
9019-001, Region 1	1.47711		
9019-001, Region 2	2.205308		
9019-001, Region 3	3.953597		

LAM - lymphangioleiomyomatosis.

Supplementary Table 2. Enriched canonical pathways for both up and downregulated genes in angiomyolipoma tumor, 621-101 and CRL 4004 cells. The enrichment analysis was done using IPA (Qiagen). Pathways were selected based on P-value cutoff <0.01.

**Ingenuity Canonical Pathways**	**-log (p-value)**	**Genes**
EIF2 Signaling	11.7	RPL32,RPL11,GRB2,RPS8,RPS23,EIF4A2,RPL7A,RPL10A,VEGFA,RPL15,FGFR3,RPS6,RPL8,RPL18A,RPS20,AKT3,RPL5,RPL37,RPL6,RPS3,RPL31,RPSA,RPLP0
mTOR Signaling	6.01	GRB2,RPS8,RPS23,VEGFB,EIF4A2,PRKCZ,VEGFA,FGFR3,RPS6,PPP2R1A,RPS20,AKT3,PPP2R5C,RPS3,RPSA
Regulation of eIF4 and p70S6K Signaling	5.74	RPS6,FGFR3,PPP2R1A,RPS20,GRB2,RPS23,RPS8,AKT3,PPP2R5C,EIF4A2,RPS3,PRKCZ,RPSA
PI3K/AKT Signaling	3.71	SYNJ2,GAB2,PPP2R1A,GRB2,AKT3,PPP2R5C,CTNNB1,PRKCZ,MCL1
p70S6K Signaling	3.53	FGFR3,GNAI2,RPS6,PPP2R1A,GRB2,PLCG2,AKT3,PPP2R5C, PRKCZ
Axonal Guidance Signaling	3.81	PLXNA1,PFN1,GNAS,GRB2,VEGFB,ADAMTS2,PRKCZ,GNAI2,VEGFA,FGFR3,ACTR3,ADAM12,GLIS1,PLCG2,ARPC2,PRKACA,AKT3,ADAM9,BMP1
Human Embryonic Stem Cell Pluripotency	3.25	FGFR3,GNAS,GRB2,TGFB2,BMPR2,AKT3,SMAD4,CTNNB1,BMP1
CREB Signaling in Neurons	3.05	FGFR3,GNAI2,GNAS,GRB2,PLCG2,ADCY3,PRKACA,AKT3,PRKCZ,EP300
Melanocyte Development and Pigmentation Signaling	3.04	FGFR3,GNAS,GRB2,PLCG2,ADCY3,PRKACA,EP300
Mouse Embryonic Stem Cell Pluripotency	2.76	FGFR3,GRB2,BMPR2,AKT3,SMAD4,DVL3,CTNNB1
Renal Cell Carcinoma Signaling	2.69	VEGFA,FGFR3,FOS,GRB2,AKT3,EP300
Role of NANOG in Mammalian Embryonic Stem Cell Pluripotency	2.42	FGFR3,GRB2,BMPR2,AKT3,SMAD4,CTNNB1,BMP1
Glioma Signaling	2.04	FGFR3,GRB2,PLCG2,AKT3,IGF2R,PRKCZ

Supplementary Table 3. Enriched biological function categories for both up and downregulated genes in angiomyolipoma tumor, 621-101 and CRL 4004 cells. Categories were selected based on P-value cutoff <0.001.

**Categories**	**Diseases or Function Annotation**	**p-value**
Cancer, Neurological Disease, Organismal Injury and Abnormalities	central nervous system cancer	2.04E-12
Cancer, Neurological Disease, Organismal Injury and Abnormalities	central nervous system tumor	3.9E-12
Cancer, Neurological Disease, Organismal Injury and Abnormalities	glioblastoma cancer	5.57E-10
Cancer, Neurological Disease, Organismal Injury and Abnormalities	gliomatosis	9.88E-10
Cell Death and Survival	neuronal cell death	2.49E-08
Cancer, Organismal Injury and Abnormalities	melanoma	7.86E-08
Cellular Development	differentiation of cells	2.63E-07
Embryonic Development, Organ Development, Organismal Development, Tissue Development	development of sensory organ	4.26E-07
Nervous System Development and Function	morphology of nervous system	2.76E-05
Cell Morphology, Cellular Assembly and Organization, Cellular Function and Maintenance, Nervous System Development and Function	extension of neurites	0.000028
Cellular Function and Maintenance, Cellular Growth and Proliferation	production of central nervous system cells	6.01E-05
Cancer, Organismal Injury and Abnormalities, Tissue Morphology, Tumor Morphology	morphology of tumor	6.79E-05
Cellular Development, Cellular Growth and Proliferation, Nervous System Development and Function, Tissue Development	proliferation of neuronal cells	0.000097
Cellular Development, Cellular Growth and Proliferation, Nervous System Development and Function, Tissue Development	growth of neurites	0.000103
Developmental Disorder, Embryonic Development, Organismal Development, Tissue Morphology	abnormal morphology of embryonic tissue	0.000123
Cancer, Neurological Disease, Organismal Injury and Abnormalities	neuroectodermal tumor	0.000133
Cancer, Organismal Injury and Abnormalities, Renal and Urological Disease	kidney carcinoma Renal Cancer and Tumors	5.74E-09<br>5.87E-09

Supplementary Table 4. Percentages of EGFP-positive cells expressing differentiation markers in *GFAP-Cre;ROSA$^{mT/mG}$ vs. GFAP-Cre; ROSA$^{mT/mG}$; Tsc1^{ff}* or in *Nestin-Cre;ROSA$^{mT/mG}$ vs. Nestin-Cre; ROSA$^{mT/mG}$;Tsc1^{ff}*

GFAP-Cre;ROSA$^{mT/mG}$ vs. GFAP-Cre; ROSA$^{mT/mG}$; Tsc1^{ff}						
	**E15.5**		**E18.5**		***Fold change in the percentage of a given subpopulation over time E15.5 vs E18.5***	
**Marker**	**EGFP+ *Tsc1$^{+/+}$***	**EGFP+ *Tsc1^{ff}***	**EGFP+ *Tsc1$^{+/+}$***	**EGFP+ *Tsc1^{ff}***	**EGFP+ *Tsc1$^{+/+}$***	**EGFP+ *Tsc1^{ff}***
EGFP	10.73	7.96	29.77	34.08	2.77	4.28
Nestin	58.98	64.57	29.45	40.54	0.50	0.63
Nestin+GFAP	1.62	3.25	25.11	12.07	15.54	3.71
Nestin+NS-tubulin	1.08	0.99	0.24	0.87	0.22	0.88
GFAP	0.08	0.25	12.71	8.61	158.32	33.92
NS-tubulin	9.25	5.79	6.39	5.42	0.69	0.94
Nestin-Cre;ROSA$^{mT/mG}$ vs. Nestin-Cre; ROSA$^{mT/mG}$;Tsc1^{ff}						
	**E15.5**		**E18.5**		***Fold change in the percentages of a given subpopulation over time E15.5 vs E18.5***	
**Marker**	**EGFP+ *Tsc1$^{+/+}$***	**EGFP+ *Tsc1^{ff}***	**EGFP+ *Tsc1$^{+/+}$***	**EGFP+ *Tsc1^{ff}***	**EGFP+ *Tsc1$^{+/+}$***	**EGFP+ *Tsc1^{ff}***
EGFP	58.30	54.13	66.62	55.27	1.14	1.02
Nestin	25.78	27.38	17.56	51.37	0.68	1.88
Nestin+GFAP	0.14	2.86	8.72	4.59	60.97	1.60
Nestin+NS-tubulin	0.78	2.32	0.21	0.75	0.27	0.33
GFAP	0.04	0.21	2.32	1.02	58.36	4.96
NS-tubulin	29.15	27.72	28.83	21.60	0.99	0.78

Values shown represent mean percentages.

GFAP-cre or Nestin-Cre, Cre recombinase under the glial fibrillary acidic protein or Nestin promoter, respectively; EGFP, enhanced green fluorescent protein; Tsc1, tuberous sclerosis complex 1; NS-tubulin, neuron-specific tubulin.
